# Identification of heavy metal pollutants and their sources in farmland: an integrated approach of risk assessment and X-ray fluorescence spectrometry

**DOI:** 10.1038/s41598-022-16177-4

**Published:** 2022-07-16

**Authors:** Xiaosong Tian, Qing Xie, Min Fan, Guanqun Chai, Guanghui Li

**Affiliations:** 1 College of Resources and Safety, Chongqing Vocational Institute of Engineering, Chongqing, 402260 China; 2 Interdisciplinary Research Center for Agriculture Green Development in Yangtze River Basin, College of Resources and Environment, Southwest University, Chongqing, 400715 China; 3 Chongqing Engineering Research Center for Soil Contamination Control and Remediation, Chongqing, 400067 China; 4 Institute of Soil and Fertilizer, Guizhou Academy of Agricultural Sciences, Guiyang, 550006 China

**Keywords:** Geochemistry, Environmental sciences

## Abstract

Investigation and assessment of farmland pollution require an efficient method to identify heavy metal (HM) pollutants and their sources. In this study, heavy metals (HMs) in farmland were determined efficiently using high-precision X-ray fluorescence (HDXRF) spectrometer. The potential ecological risk and health risk of HMs in farmland near eight villages of Wushan County in China were quantified using an integrated method of concentration-oriented risk assessment (CORA) and source-oriented risk assessment (SORA). The CORA results showed that Cd in farmland near the villages of Liuping (LP) and Jianping (JP) posed a “very high” potential ecological risk, which is mainly ascribed to soil Cd (single potential ecological risk index (${E}_{r}^{i}$) of Cd in villages LP and JP, ${E}_{r}^{i}$ = 2307 and 568 > 320). A “moderate” potential ecological risk was present in other six villages. The overall non-carcinogenic risk (hazard index (HI) = 1.2 > 1) of HMs for children in village LP was unacceptable. The contributions of HMs decrease in the order of Cr > As > Cd > Pb > Ni > Cu > Zn. The total carcinogenic risk (TCR = 2.1 × 10^–4^ > 1.0 × 10^–4^) of HMs in village LP was unacceptable, with HMs contributions decreasing in the order of Cr > Ni > Cd > As > Pb. Furthermore, three source profiles were assigned by the positive matrix factorization: F1: agricultural activity; F2: geological anomaly originating from HMs-rich rocks; F3: the natural geological background. According to the results of SORA, F2 was the highest contributor to PER in village LP, up to 64.4%. Meanwhile, the contributions of three factors to HI in village LP were 19.0% (F1), 53.6% (F2), and 27.4% (F3), respectively. It is worth noting that TCR (1.2 × 10^–4^) from F2 surpassed the threshold of 1.0 × 10^–4^, with an unacceptable carcinogenic risk level. As mentioned above, the HM pollutants (i.e., Cd and Cr) and their main sources (i.e., F2) in this area should be considered. These results show that an integrated approach combining risk assessments with the determination of HM concentration and identification of HM source is effective in identifying HM pollutants and sources and provides a good methodological reference for effective prevention and control of HM pollution in farmland.

## Introduction

Recently, the enrichment of heavy metals (HMs) in farmland has become a matter of great concern globally due to the ubiquity, persistence, and toxicity of these pollutants^1–3^. Since the release of the *National Soil Pollution Survey Bulletin of China* in 2014, governments and researchers have paid much more attention to the remediation and risk control of HM-polluted farmland^4^. In designing appropriate management policies, the first task is to determine concentration of HMs and evaluate soil contamination level by the multi-step wet-chemistry methods^5^. Although these techniques are highly accurate, they are costly and time-consuming^6^, which limits large-scale farmland soil investigations^7,8^. Researchers have recently focused on the use of X-ray fluorescence (XRF) spectrometer, which is non-destructiveness, can detect multiple elements capability, is easy to use, rapid, and low cost^6,9,10^. They have been successfully applied to the determination of HMs in the investigation of contaminated soils in and around industrial sites^10–14^. For example, Jiang, et al.^15^ used XRF to quantify Cr, Zn, Pb, Cu, and Ni concentrations in soil for health risk assessment. Furthermore, high-precision X-ray fluorescence (HDXRF) spectrometry can reliably determine low HM concentrations in farmland, such as that of Cd^16,17^. This provides the technical means for high-efficiency investigation and assessment of HM-polluted farmland.

To systematically evaluate the characteristics of HM pollution, risk assessments and source identifications of HMs in soil have been increasingly used^18–20^. The potential ecological risk (PER) and health risk (HR) assessment methods have been applied to assess the threats of HMs to the environment and human body, respectively^21,22^. HR and PER assessments based on reliable HM concentrations provide critical references for establishing the corresponding soil remediation and risk management policies^19,23^. The HM pollutants in farmland can be identified by HR and PER methods. Notably, determining HM source categories in farmland and their corresponding contributions is of great value in designing work plans for soil remediation and risk management. Source identification and apportionment were common measures for better understanding the characteristics of HM-contaminated soils^20,23^.

Multivariate statistical analysis methods (such as principal component analysis, multi-linear regression, hierarchical cluster analysis) and geostatistical analysis have been widely adopted to identify the sources of soil HMs^21,24^. Both multivariate and geostatistical analyses can roughly identify the number and type of sources and lack the potential to assess HM source contributions^19^. Therefore, quantitative identification of possible sources of HMs in soils is quite important for controlling and reducing pollution. The quantitative approaches to source apportionment mainly include the positive matrix factorization (PMF) model, CMB model, UNMIX model, and PCA-APCS model^15,22^. In multitudinous source apportionment methods, the PMF model, which was recommended by the U.S. Environmental Protection Agency (USEPA), is one of the most common approaches to quantifying the contributions of different pollution sources^20,23,25^. The PMF model can successfully perform pollution source apportionment in various ambient media, such as particulate matter, sediments, and soils^3,26,27^, and is also an efficient tool for local authorities in formulating pollution prevention and risk control measures^23,28,29^. Nevertheless, risk identification or source apportionment has been performed individually in numerous studies^20,30^, and only a few have focused on the combinations of the PMF model with risk assessment models (such as PER and HR models)^15,19,22,31^. For example, Jiang, et al.^22^ used an integrated approach (including geostatistics, the PMF model, and a risk assessment model) to identify and quantify the sources of soil HMs in woodland, construction land, and farmland. Therefore, it is essential to combine the PMF model and risk assessment models to identify HM pollutants and sources based on concentration/source-oriented potential ecological risk and health risk assessment^3,19^.

However, the use of such integrated approaches to inform HM risk management in farmland remain rare, especially in term of large-scale pollution investigations. Accordingly, an integrated approach that is non-destructiveness, easy to use, rapid, and low-cost is needed. The present study uses an integrated method based on the concentrations-oriented risk assessment (CORA) and sources-oriented risk assessment (SORA) approach and the use of an HDXRF spectrometer to perform the risk assessment and source apportionment. The primary objectives of coupling the CORA/SORA approach with the HDXRF spectrometer include three aspects: (1) providing a high-efficiency, environmentally friendly, low-cost, and rapid method for investigating HM pollution status and potential risk in farmland based on the HDXRF spectrometer dataset; (2) clarifying HM pollutants and pollution level according to the CORA approach; and (3) providing quantitative indications of HM sources based on the SORA approach. The ultimate aim of this study is to provide crucial information for low-cost and efficient risk management and risk control for farmland.

## Materials and methods

### Study area

The study area was located in Wushan County of southwestern China (31° 31'‒30° 58' N, 109° 50'‒109° 58' E; altitude = 500–1500 m; Fig. S1). This area has a humid subtropical monsoon climate with an average annual precipitation of 1052.4 mm and an average temperature of 18.0 °C. The outcropping rocks in this area include lithologies from the Silurian to the Permian periods, primarily composed of limestone, siltstone, black shale, and coal seams^32,33^. The study area includes eight villages: Liu Ping (LP), Jian Ping (JP), Chun Xiao (CX), Huang Yan (HY), Qing Tai (QT), Yun Tai (YT), Zhong Huo (ZH), and Gua Piao (GP). The area is a rural residential area and dryland agricultural planting area. The main soil types are lime soil and yellow soil. Before the 1970s, there was coal mining activity in the northeast of the study area. Tang, et al.^32^ found that the concentration of Cd in arable soils (1.01‒59.7 mg/kg) was significantly higher than the soil background value and greatly exceeded the risk screening value (0.3‒0.6 mg/kg) and intervention value (1.5‒4.0 mg/kg) according to standard GB15618-2018**.** In this area, Cd is an essential factor in inducing environmental and health problems^33,34^. However, the contributions of each HMs and each source remain unclear.

### Sample collection, preparation, and test

In September 2019, 90 soil samples were randomly collected in farmland from eight villages to identify HM pollutants and conduct source apportionment in Fig. S1. The coordinates of sampling sites were recorded by using a portable global positioning system (GPS; UniStrong, A5)^11^. The spatial distribution map of samples was created in ArcGIS version 10.0 (http://www.esri.com/). Each sample was a mixture of subsamples located within a distance of about 10 m in the same field^12^. After getting rid of the grass, roots, stones, and other non-soil material, 1 kg soil samples were placed in sealed polythene bags. They were taken to the laboratory for air-dried at room temperature^6,35^. The air-dried samples were ground using a stick on the brown paper and passed through a 100-mesh nylon sieve^9,36^ to improve the accuracy of XRF tests. The concentrations of HMs (including Cr, Ni, Cu, Zn, As, Cd, and Pb) were determined by HDXRF spectrometer (Cadence, XOS, USA)^16,17^. Before the determination process, soil samples were put into a sample cup and compacted with a pestle. The test procedure was performed for 10 min. During the test process, the sample cup film was replaced between each measurement to minimize or avoid the effect of cross-contamination between samples. Soil pH value was measured with a pH meter (Hach H160NP), which was placed in the suspension at 1:2.5 soil to water ratio^12,37^.

### Quality assurance

During the testing process, the certified reference materials (GSS4 and GSS5) were tested to confirm the precision and accuracy of HDXRF spectrometer analysis at intervals of 10‒20 soil samples. The recoveries of Cr, Ni, Cu, Zn, As, Cd, and Pb ranged from 90 to 110%. The precisions of the HDXRF spectrometer for Cr, Ni, Cu, Zn, As, Cd, and Pb were acceptable, with relative standard deviations (RSD) of < 10%. The HDXRF spectrometer recoveries and precisions of each HM were shown in Table S1. Moreover, the limits of detection (LODs) of Cr, Ni, Cu, Zn, As, Cd, and Pb were 16, 4, 0.8, 1.6, 0.8, 0.09, and 0.8 mg/kg, respectively, which meet the standard for the investigation of soil environmental quality in GB15618‒2018.

### Identifications of the pollutant and source

**Figure 1:**
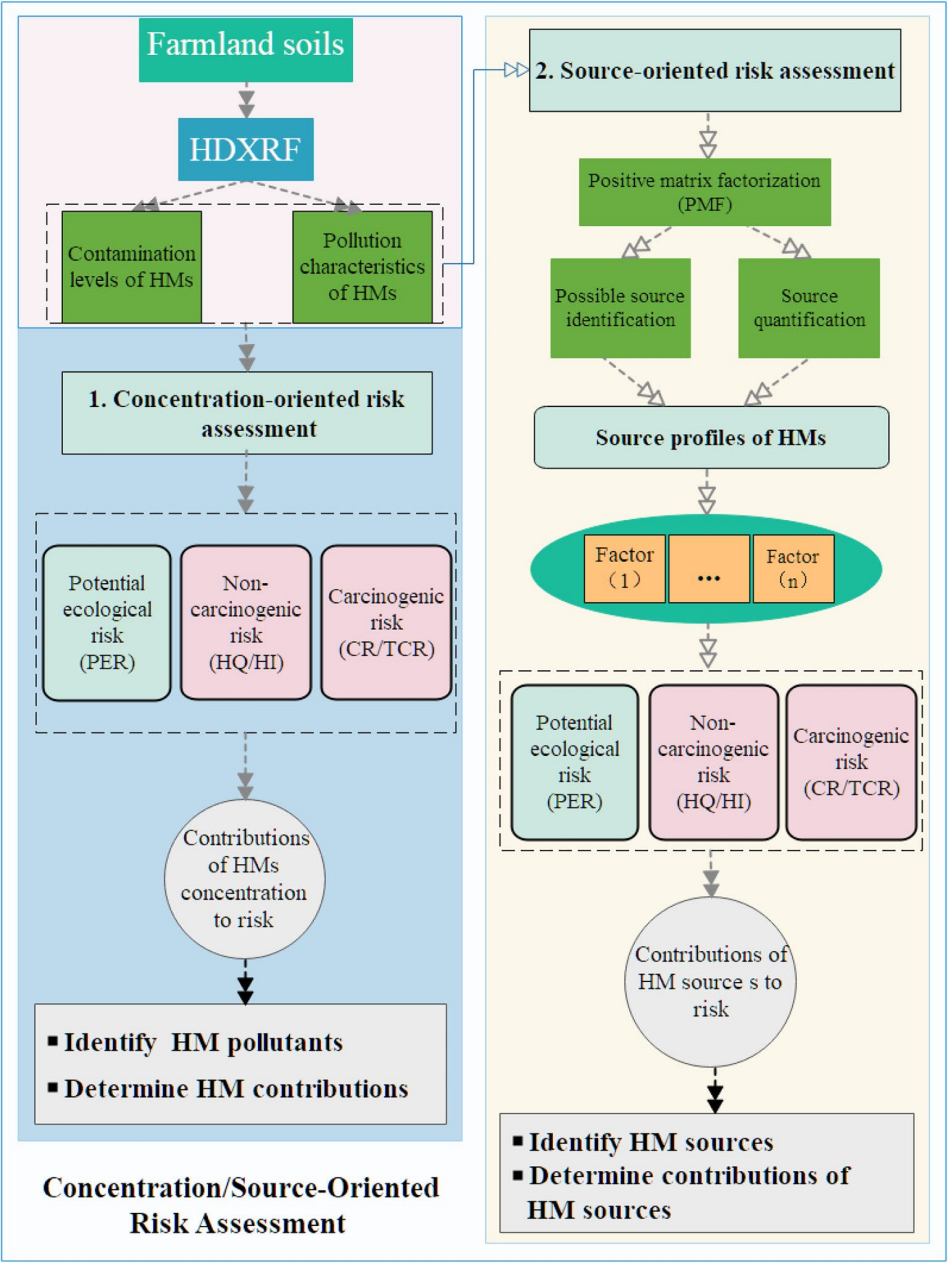
Framework of the concentration/source-oriented risk assessment approach used in this study.

The approach consisted of two risk assessment systems: (1) HM concentrations (concentration-oriented risk assessment, CORA) and (2) source apportionment (source-oriented risk assessment, SORA). The implementation of this approach is described in Fig. 1. According to the integrated approach, CORA and SORA were used to identify HM pollutants and sources, respectively.

#### Potential ecological risk

PER is a quantitative index combining ecological and toxicological factors to evaluate the individual or comprehensive ecological effects of HMs. It can be defined as per Eq. (1)^38^:

1 $$PER=\sum {E}_{r}^{i}=\sum {T}_{r}^{i}\times \left(\frac{{C}_{s}^{i}}{{C}_{n}^{i}}\right)$$

where ${E}_{r}^{i}$ represents a single potential ecological risk index, ${T}_{r}^{i}$ represents the metal’s toxic response factor (Zn = 1; Cr = 2; Cu, Pb, Ni = 5; As = 10, and Cd = 30)^39^. ${C}_{s}^{i}$ is the concentration of *i*th element in the soil (mg/kg), ${C}_{n}^{i}$ is the soil background value of the Three Gorges Reservoir (TGR) in China^40^. $PER$ is the sum of ${E}_{r}^{i}$. Table S2 shows the classifications of ${E}_{r}^{i}$ and $PER$ values^41^.

#### Health risk

The health risk assessment model developed by the USEPA has been used to evaluate non-carcinogenic and carcinogenic effects on humans. Compared to adults, children may be a more sensitive exposure group because of their behaviors (such as finger sucking)^42^. This characteristic is often regarded as a critical exposure pathway for soil HMs in the soil in children^1,43^. This study performed the health risk assessment related to children. Usually, exposure of humans to soil HMs has three potential pathways: (1) incidental soil ingestion, (2) direct dermal contact, and (3) soil vapor inhalation^11^. The average daily intake (mg/(kg day)) of the ith HM through soil ingestion (${\mathrm{ADI}}_{i,ing}$), dermal contact (${ADI}_{i,der}$), and inhalation (${ADI}_{i,inh}$) from the same soil sample can be evaluated by Eqs. (2)–(4)^44^:

2 $${ADI}_{i,ing}=\frac{{C}_{i,s}\times {IR}_{i,ing}\times EF\times ED}{BW\times AT}\times {10 }^{-6}$$

3 $${ADI}_{i,der}=\frac{{C}_{i,s}\times SA\times AF\times ABS\times EF\times ED}{BW\times AT}$$

4 $${ADI}_{i,inh}=\frac{{C}_{i,s}\times {IR}_{i,inh}\times EF\times ED}{PEF\times BW\times AT}$$

where ${C}_{i,s}$ represents the concentration of the *i*th HM in the soil sample (mg/kg). ${IR}_{i,ing}$ and ${IR}_{i,inh}$ represent the daily ingestion (mg/day) and inhalation (m^3^/day) rates of soil, respectively. EF is the exposure frequency (days/year); ED is the exposure duration (years); BW is the body weight of the exposed individual (kg); AT is the average time exposure to the contaminated soil (day); SA is the exposed surface area of the skin (cm^2^); AF is the skin adherence factor (kg/ (cm^2^ day)); ABS is the dermal absorption factor (Unitless); and PEF is the emission factor (m^3^/kg). The detailed parameters are shown in Table S3.

Based on the results of the average daily intake dose of the ***i***th metal, hazard quotients (${HQ}_{i}$) were utilized to assess the non-carcinogenic risk. For soils contaminated by multiple HMs, a hazard index (*HI*) was applied to determine the overall non-carcinogenic risk using Eqs. (5) and (6)^19,44,45^:

5 $${HQ}_{i}=\frac{{ADI}_{i, ing}}{{RfD}_{i, ing}} +\frac{{ADI}_{i, ider}}{{RfD}_{i, der}}+\frac{{ADI}_{i, inh}}{{RfD}_{i, inh}}$$

6 $$HI=\sum_{i=1}^{j}{HQ}_{i}$$

where ${RfD}_{i, ing}$, ${RfD}_{i, der}$, and ${RfD}_{i, inh}$ are the reference exposure doses of *i*th HM (mg/(kg day)) via soil ingestion, dermal contact, and inhalation, respectively, as shown in Table S4. The non-carcinogenic health effect is not considered to be serious when ${HQ}_{i}$ and *HI* are < 1.

Moreover, the carcinogenic risk (${CR}_{i}$) of the *i*th carcinogenic element and total carcinogenic risk (TCR) of multiple HMs in contaminated soils can be evaluated by Eqs. (7) and (8)^19,44^:

7 $${CR}_{i}={ADI }_{i,ing}\times {SF}_{i,ing}+{ADI }_{i,der}\times {SF}_{i,der}+{ADI }_{i,inh}\times {SF}_{i,inh}$$

8 $$TCR=\sum_{i}^{j}{\mathrm{CR}}_{i}$$

where ${SF}_{i,ing}$, ${SF}_{i,der}$,${SF}_{i,inh}$ are the carcinogenic slope factors (per mg/(kg $\times$ day)) for *i*th HM via soil ingestion, dermal contact, and inhalation, respectively, and the detailed parameters are shown in Table S4. Generally, ${CR}_{i}$ or $TCR$ > 1 × 10^−4^ are considered to represent a significant cancer risk; 1 × 10^−6^ < ${CR}_{i}$ or $TCR$ < 1 × 10^−4^ are considered acceptable; and ${CR}_{i}$ or $TCR$< 1 × 10^−6^ are negligible^19,43^.

#### Positive matrix factorization

The PMF is a modified factorization method that the USEPA has recommended for source apportionment and was developed by Paatero and Tapper^25^. In this study, EPA-PMF (version 5.0) was adopted to apportion the dominant sources of HMs in the soil samples. The purpose of the PMF was to address source profiles and source contributions based on composition datasets, as shown in the following Eq. (9):

9 $${x}_{ij}=\sum_{k=1}^{p}{(g}_{ik}{f}_{kj}+{e}_{ij})$$

Source profiles and factor contributions were computed by minimizing the objective function Q (Eq. (10)):

10 $$Q=\sum_{i=1}^{n}\sum_{j=1}^{m}{\left[\frac{{x}_{ij}-\sum_{k=1}^{p}{g}_{ik}{f}_{kj}}{{u}_{ij}}\right]}^{2}=\sum_{i=1}^{n}\sum_{j=1}^{m}{\left(\frac{{e}_{ij}}{{u}_{ij}}\right)}^{2}$$

The uncertainty in the concentrations of the various HMs was calculated using Eq. (11):

11 $${\text{u}}_{{{\text{ij}}}} = \left\{ {\begin{array}{*{20}c} {\frac{5}{6} \times {\text{MDL}}, ({\text{c}} \le {\text{MDL}})} \\ {\sqrt {({\text{s}} \times {\text{c}})^{2} + (0.5 \times {\text{MDL}})^{2} } , ({\text{c}} > {\text{MDL}})} \\ \end{array} } \right.$$

where *x*_*ij*_ is the concentration matrix of the *j*th HM in the *i*th sample; *g*_*ik*_ is the contribution matrix of the *k*th source factor to the *i*th sample; *f*_*kj*_ is the source profile of the *j*th HM for the *k*th source factor; *e*_*ij*_ is the residual of each HM; *u*_ij_ is the uncertainty in the *j*th HM of the *i*th sample; *s* is the relative standard deviation; *c* is the concentration of a specific HM; and MDL is the method detection limit.

## Results and discussion

### Descriptive statistics of HMs in farmland

Table 1 shows the descriptive statistics of HMs in farmland. The soil pH values ranged from 4.75 to 8.47. The mean concentrations of Cr (155.33 mg/kg), Cu (46.53 mg/kg), As (16.61 mg/kg), Cd (3.88 mg/kg), Pb (32.36 mg/kg), Ni (53.22 mg/kg), and Zn (119.16 mg/kg) exceeded the background values for soils in the TGR by 0.99-, 0.86-, 1.85-, 28.82-, 0.36-, 0.81-, and 0.71-fold, respectively. Furthermore, the mean concentrations of Cr (155.33 mg/kg) and Cd (3.88 mg/kg) surpassed the risk screening values (RSV*)* of other land-use types at the corresponding soil pH according to GB15618-2018 (Table S5). Compared with HM concentrations in agricultural soils in other regions of China, such as the Hexi corridor^23^, Taiyuan^46^, Wenling^19^, Jiaxing^47^, and Tianjin^3^, those of Cr and Cd in this area were much higher. These results suggested that soil HMs in this study area might pose a higher potential ecological risk for the surrounding environment or health risk for residents.

**Table 1:** Descriptive statistics of HM concentrations (mg/kg) in soils.

Region	Parameter	Heavy metals						
		Cr	Cu	As	Cd	Pb	Ni	Zn
This study	N	90	90	90	90	90	90	90
	Mean	155.33	46.53	16.61	3.88	32.36	53.22	119.16
	CV	1.13	0.44	0.19	1.90	0.08	0.64	0.50
	Min	56.05	20.65	9.68	< 0.27	27.05	22.35	66.85
	Max	1106.70	117.75	27.40	38.95	42.45	227.50	383.60
	Skewness	3.58	1.19	0.30	2.92	0.94	2.64	2.79
	Kurtosis	14.30	1.45	0.39	8.78	2.52	8.73	8.65
	*C*_*n*_	78.03	25.00	5.83	0.13	23.88	29.47	69.88
Hexi corridor^19^	Mean	97.51	35.20	‒	–	5.54	47.42	75.34
Taiyuan^49^	Mean	74.10	32.11	10.70	0.25	27.87	29.74	90.76
Wenling^20^	Mean	74.78	52.59	10.25	0.34	33.84	35.03	143.74
Jiaxing^50^	Mean	87.80	32.40	8.55	0.22	33.90	36.40	94.90
Tianjin^3^	Mean	69.40	29.10	9.35	0.27	26.70	32.30	105.40

*SD* standard deviation, *CV* coefficient of variance, *C*_*n*_ background values of soil HMs in the TGR^40^.

According to the coefficient of variation (CV) of seven HMs, we can infer that HMs in this area presented three kinds of distribution patterns. The first group included As and Pb, the second group consisted of Cu, Ni, and Zn, and the third group included Cr and Cd. The CV of Cr (1.13) and Cd (1.90) varied considerably (Table 1), indicating that the spatial distribution of the two HMs were heterogeneous. A lower CV and accumulation of Cu, As, Pb, Ni, and Zn might reflect a slight disturbance by human activities^19^. The difference in agricultural soil quality might be related to the interference of human activities or geological anomalies^3,30^.

Some reports indicated that HM sources in agricultural soils are mainly affected by anthropogenic input^11,19^, such as wastewater irrigation, sludges application, fertilizers, and agrochemicals^11^. However, a high geochemical background may also be a crucial source of HMs in soils in specific areas^48,49^. A multi-objective regional geochemical investigation in China found high Cd anomalies in the Yangtze River basin, especially its upper reaches^34,50^. Earlier research has reported high Cd concentration in farmland due to the geogenic sources of black shales and coal mining activities^32,51^. Previous studies have found that soil HMs in high-geochemical background areas present a high potential risk to the surrounding environment and its residents in this area^33,52^. Liu, et al.^49^ also indicated that the soil-rice systems in high-geochemical-background areas are in a poor state of health. Combined with pollution investigation in the field, the study area is a typical agricultural production area, the primary source of HMs pollution in this area may be a geological anomaly, agricultural activities, and traffic emissions from the county road.

### Identification of HM pollutants based on CORA

#### Potential ecological risk

**Figure 2:**
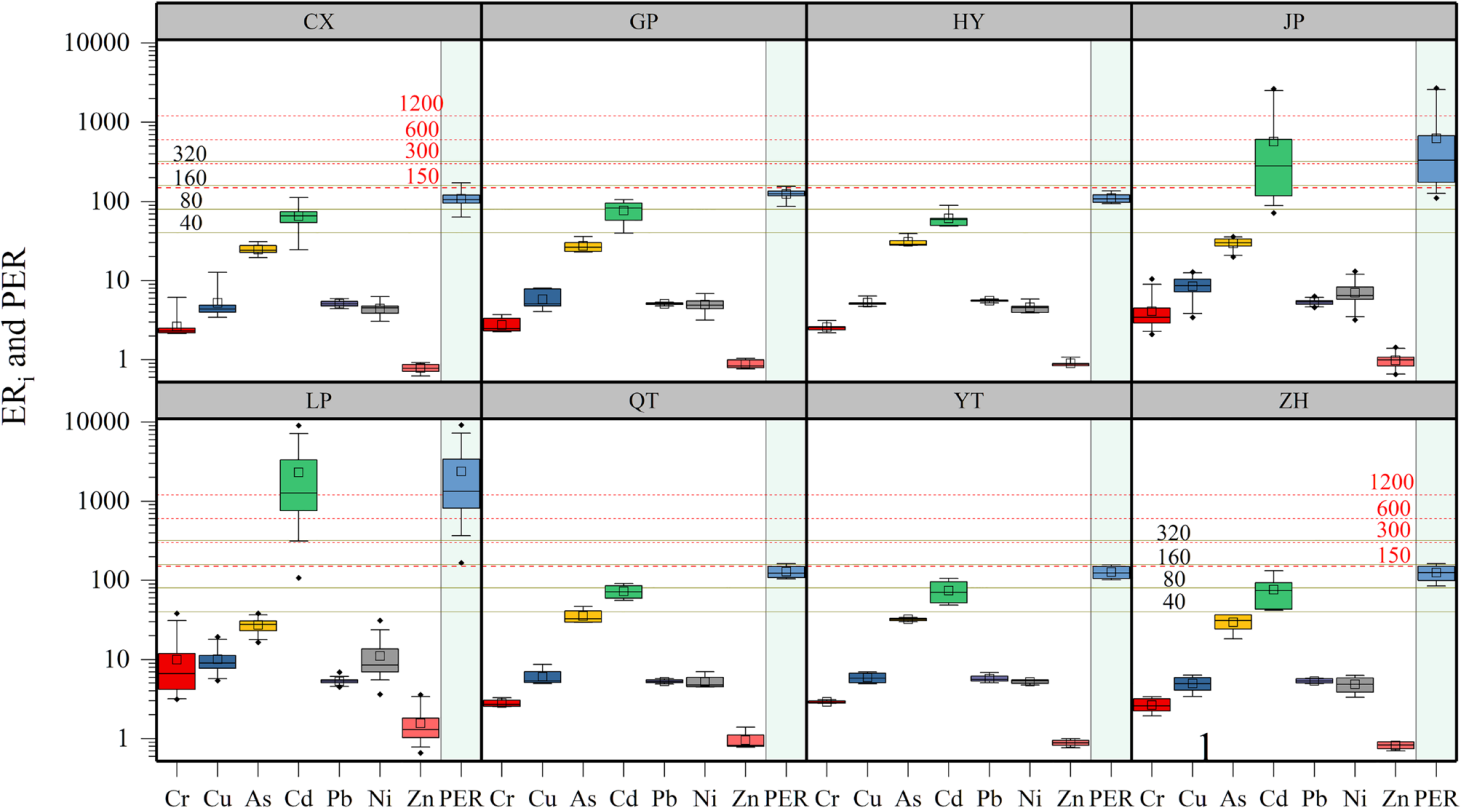
ER_i_ and PER of HMs in various villages. Green horizontal reference lines represent the corresponding classification criteria of ${E}_{r}^{i}$ (40, 80, 120, 320); Red horizontal reference lines represent the corresponding classification criteria of PER (150, 300, 600, 1200). Boxes represent the 25th and 75th percentiles, the whiskers represent the 5th and 95th percentiles, and the hollow squares represent means.

The PER of soil HMs to the environment was computed to identify the HM pollutants. Variable ${E}_{r}^{i}$ and $PER$ are illustrated in Fig. 2. The mean ${E}_{r}^{i}$ values of Cr, As, Cu, Ni, Pb, and Zn in farmland in the eight villages were < 40, indicating that six elements presented low potential ecological risk to the surrounding environment. These were consistent on a national scale in China^41^. The mean ${E}_{r}^{i}$ values of Cd in farmland in villages CX, GP, HY, QT, YT and ZH were 66, 77, 61, 72, 74, and 76, respectively, representing “moderate” risk. The ${E}_{r}^{i}$ of Cd in farmland in villages LP and JP were 2307 and 568, respectively, representing “very high” risk. The mean PER values of HMs in villages CX, GP, HY, JP, LP, QT, YT, and ZH were 109, 125, 111, 624, 2372, 128, 127, and 124, respectively, indicating that farmland HMs in villages JP and LP represented “moderate” and “very high” risk, respectively. Furthermore, the contributions of HMs to the PER had notable differences, and are ranked in descending order as Cd > As > Ni, Cu, Cr, and Pb > Zn. It is worth noting that the farmland around village LP posed a “very high” risk level, while Cd made the greatest contribution to the PER. The primary reasons are the high concentration and low background value of Cd in soils^32^, and the increased contribution rate of the monomial PER index of Cd^41^. Cadmium should be considered the worst HM pollutant based on the PER assessment.

#### Health risk

**Figure 3:**
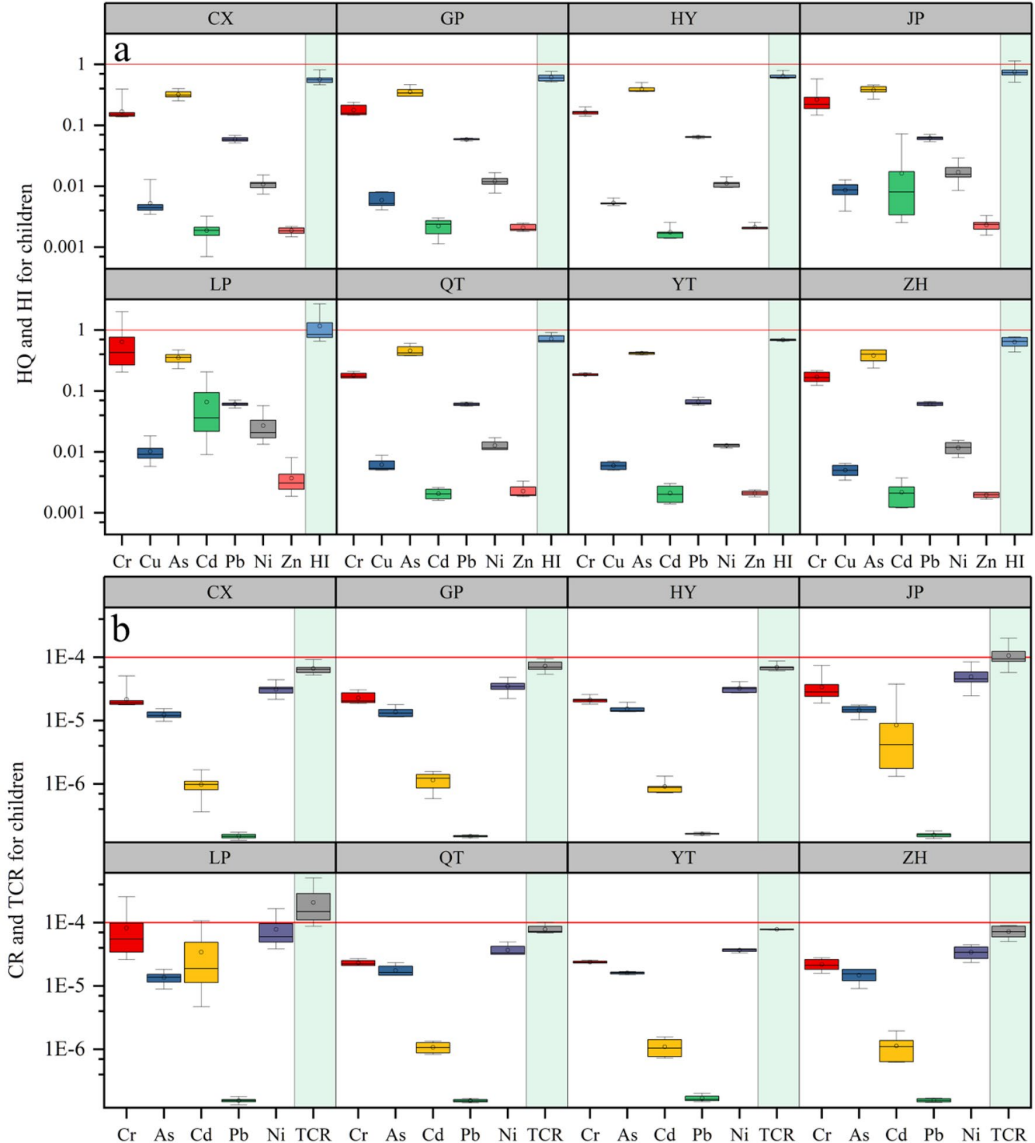
HQ and HI of HMs related to children (**a**), CR and TCR of HMs related to children (**b**), in eight villages. Red horizontal reference lines represent the corresponding classification criteria (1 for HQ and HI, and 1.0 × 10^–4^ for CR and TCR). Boxes represent the 25th and 75th percentiles, whiskers represent the 5th and 95th percentiles, and the hollow circles represent means.

As illustrated in Fig. 3a, the mean HQ and HI values for children in villages CX, GP, HY, JP, QT, YT, and ZH were below 1; while the mean HI values for children in village LP were 1.2, which exceeds the guideline value of 1, indicating that the non-carcinogenic risk of HMs to children in village LP was unacceptable. The mean HQs of HMs in village LP are ranked in descending order as Cr > As > Cd > Pb > Ni > Cu > Zn, while those in village JP are As > Cr > Pb > Ni > Cd > Cu > Zn, and those in other villages are As > Cr > Pb > Ni > Cu > Cd and Zn. Notably, the Cr made the greatest contributions to the HQ (non-carcinogenic risk) of soil HMs in village LP. This might be ascribed to the low RfD and high Cr concentration in soils^1^. Soil HMs in village LP posed an unacceptable non-carcinogenic risk to children’s health. The main reason is that children are likely to have a higher HM ingestion rate due to their unique behaviors (e.g., finger sucking^42^, which is regarded as one of the critical exposure pathways for children^1,43^). Another reason is that children have less weight than adults, so there is a higher susceptibility of exposure to environmental contaminants per unit of body weight^3^.

The carcinogenic risks of HMs to children were estimated, as shown in Fig. 3b. The CR and TCR of each HM to children in various villages were estimated. Mean CRs in all villages (except in village LP) were below the guideline value of 1.0 × 10^–4^, gradually decreased in the order of Ni > Cr > As > Cd > Pb; and mean CR values for children in LP village were also below the guideline value of 1.0 × 10^–4^, and gradually reduced in the order of Cr > Ni > Cd > As > Pb. The mean TCRs of HMs to children in villages CX, GP, HY, QT, YT, and ZH were below the guideline value (1.0 × 10^–4^), while those in villages LP (2.1 × 10^–4^) and JP (1.1 × 10^–4^) exceeded the guideline value, indicating that the carcinogenic risks of agricultural soils HMs in villages LP and JP were unacceptable^19,43^. The carcinogenic risk level of soil HMs in village LP was higher than in the other villages, which is attributable to the higher concentrations of Cr and Ni in nearby farmland and should be noticed. This might be attributable to a short exposure duration for children and high HM concentrations^42^. Combining HI with TCR, we could conclude that Cr and Ni are HMs contributing most to the non-carcinogenic and carcinogenic risks in this area. The village LP as a hotspot requires risk management and remediation of contaminated soils.

### Identification of HM sources based on SORA

#### HM source apportionments

To further identify the HM sources in farmland and determine their contributions, a PMF was conducted. During the computational process, the number of factors was set to 2, 3, and 4, and 20 PMF runs were used. In changing the factors from 2 to 4, a successive decrease in Q_robust_/Q_expected_ was found (Table S6). The reduction in Q_robust_/Q_expected_ was much more minor with the factor changes from 3 to 4, indicating that three factors may be the optimal solution for explaining HM sources, at which time most of the residual was ranged from -3 to 3. Furthermore, the decrease in Q (DISP%dQ) of < 0.1% indicates that the PMF results were acceptable^3,53^. The signal-to-noise ratios (S/N) of HMs were > 2, which is categorized as strong, ensuring the rationality of the model. The coefficient (*R*^2^) between the observed and predicted concentrations of HMs ranged from 0.51 to 0.98 (Table S7), suggesting a strong correlation between them. Therefore, this model is suitable for explaining the information contained in the initial data.

**Figure 4:**
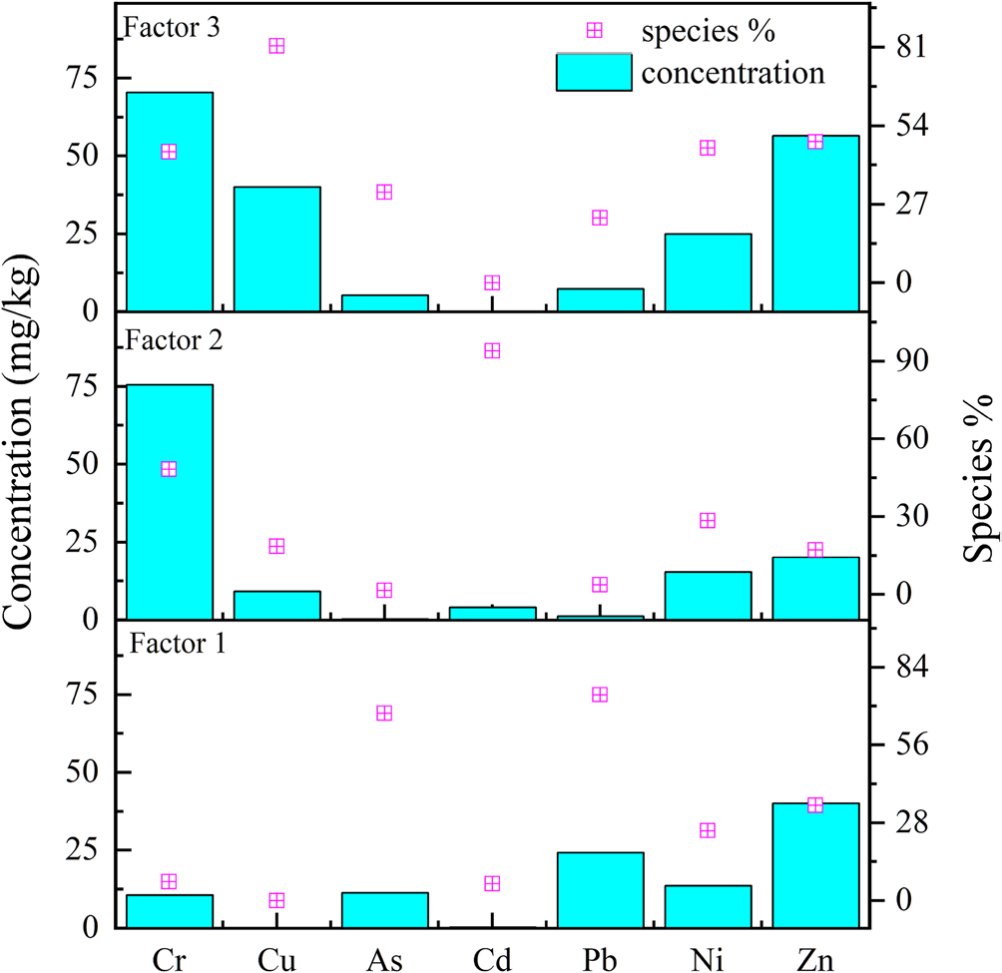
Fractional concentrations and factor contributions of (n = 90).

Factor 1 (F1) was predominated by Pb (74.1%), As (67.4%), and Zn (34.3%); their concentrations originating from F1 were 24.12, 11.26, and 40.1 mg/kg, respectively (Fig. 4). The Pb and Zn concentrations were close to the background values of the TGR, while the As concentration surpassed the background values (5.83 mg/kg). The low concentration and CV of As in farmland indicate that F1 was related to agricultural activity (non-point source pollution), such as agrochemical application, which contributes to As accumulation in farmland^19^. The research reported that the increased As concentration in farmland in China mainly originates from the application of pesticides, inorganic fertilizers, and livestock manure^24^. Pb is the primary indicator of traffic emission. It is associated with lead-acid batteries, catalysts and fuel combustion, while Zn comes from the corrosion of galvanized parts and the tires wear^15,31^. And other reports also suggested that the loss of tires and other vehicle components can easily lead to the accumulation of Zn and Ni^54^. Based on the above, F1 was defined as a “mixed source” that included agriculture and traffic emissions.

Factor 2 (F2) was mainly loaded by Cd (accounting for 94.0% of the total Cd in soils), and its corresponding concentration was 3.94 mg/kg, which exceeds the background value and even RIV in Table S4. F2 also included Cr (48.3%) and Ni (28.5%). The high CV values of Ni, Cr, and Cd also indicate apparent spatial heterogeneity in their concentrations. Compared with the Cd-rich local coal and black shale in this area^32,34^, the high-Cd- and -Cr-concentration F2 source profile can be ascribed to the high Cd geological background and historical mining activity. Past mining and natural weathering could further increase the Cd release from HM-rich rocks (e.g., black shale and Cd-rich local coal)^19,55^. Thereby, F2 was identified as a geological anomaly from the HM-rich rocks in the high-Cd geological background area.

Factor 3 (F3) was dominated by Cu (81.4%), Zn (48.5%), Ni (46.4%), Cr (45.0%), As (32.2%) and Pb (22.3%), which had the corresponding fractional concentrations of 39.94, 56.53, 24.94, 70.38, 5.20 and 7.26 mg/kg, respectively. These are close to the background values and below the RIVs in Table S4, indicating that soils HMs had no accumulation from this source^31^. Some studies have also concluded that Cr and Ni in farmland mainly come from the parent material^19,23,30^. Jiang, et al.^31^ and Cai, et al.^56^ also reported that Cr, Ni, and Co are related to the soil parent material. Previous research identified that Pb and Cu concentrations in soils can be ascribed to the parent material and pedogenic processes^34^. These results infer that F3 mainly had a natural geological background origin. Thereby, F3 was defined as the natural geological background.

#### HM source contributions

**Figure 5:**
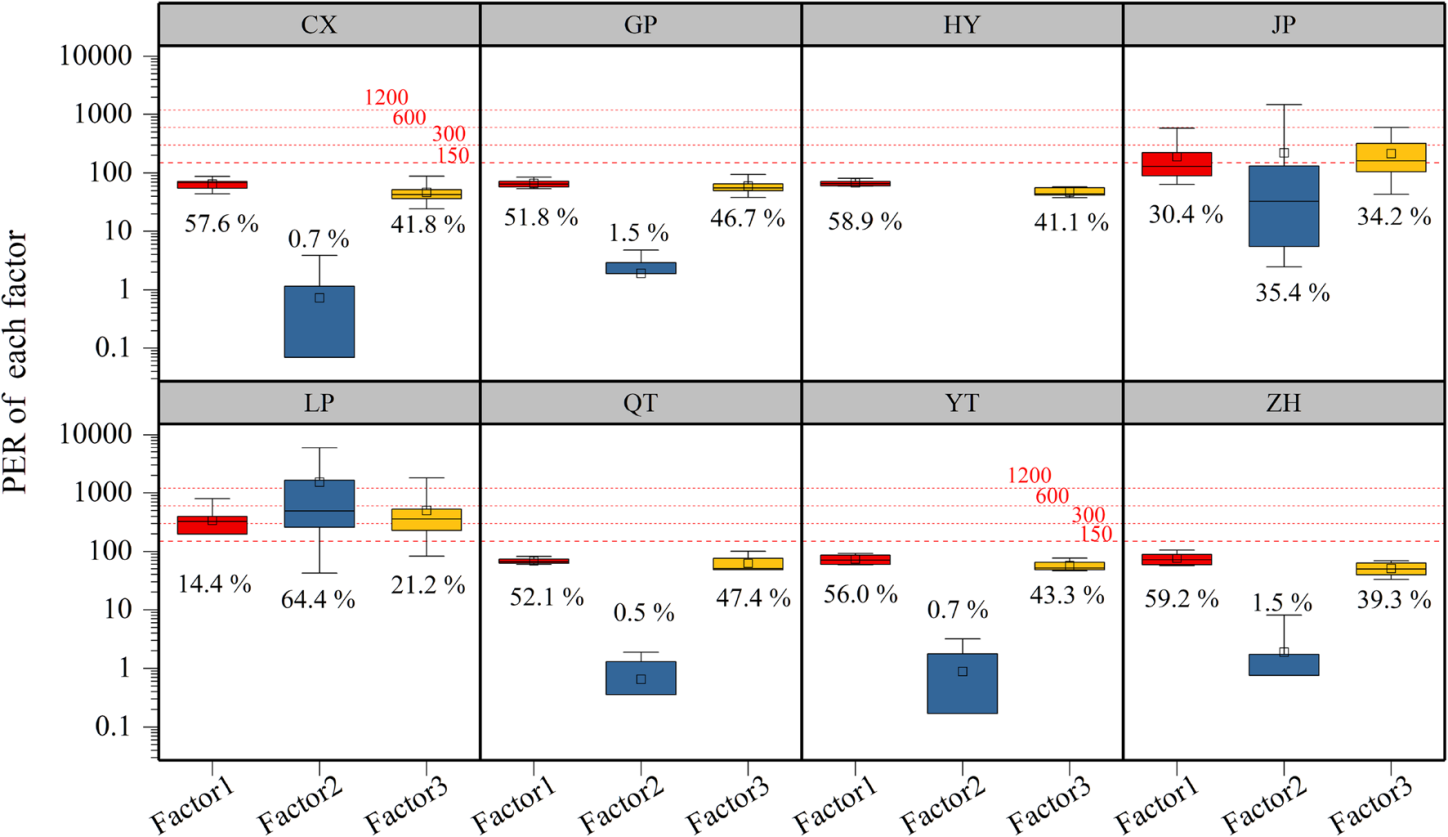
Contributions of various sources to PER. The red horizontal reference lines represent the corresponding classification criteria of PER (150, 300, 600,1200). Boxes represent the interquartile range (i.e., 25th and 75th percentiles), the hollow squares represent the average values, and the whiskers represent the 5th and 95th percentiles.

A PMF-based PER model was established to quantify the potential ecological risks of soil HMs from the three identified sources (Fig. 5). Similar contributions of F1, F2, and F3 to PER were observed in villages CX, GP, HY, QT, YT, and ZH, about 51.8‒59.2%, 0‒1.5%, and 39.3‒47.4%, respectively. Meanwhile, the mean PER of each factor did not exceed the classification criterion of 150 in villages CX, GP, HY, QT, YT, and ZH, indicating that there was no potential ecological risk from these sources. The associated PERs of HMs and source apportionment indicate an acceptable potential ecological risk in these villages. The contributions of F1, F2, and F3 to PER in village JP were 30.4%, 35.4%, and 34.2%, respectively, and the mean PERs were 190, 221, and 210, respectively, demonstrating that each factor represents a “general” potential ecological risk. Besides, the contributions of F1, F2, and F3 to PER in village LP were 14.4%, 64.4%, and 21.2%, respectively, and the mean PERs were 340, 1519, and 500, respectively. It is worth noting that the PER of F2 contribution reached the “very high” level in village LP because of the high potential ecological risk index of Cd^41^ and its high concentration load in F2 due to the release or weathering of Cd-rich rocks and coals^32^. Similar results have been observed in other studies^22^. Therefore, we should pay much more attention to F2 according to the PER inferred by source apportionment.

**Figure 6:**
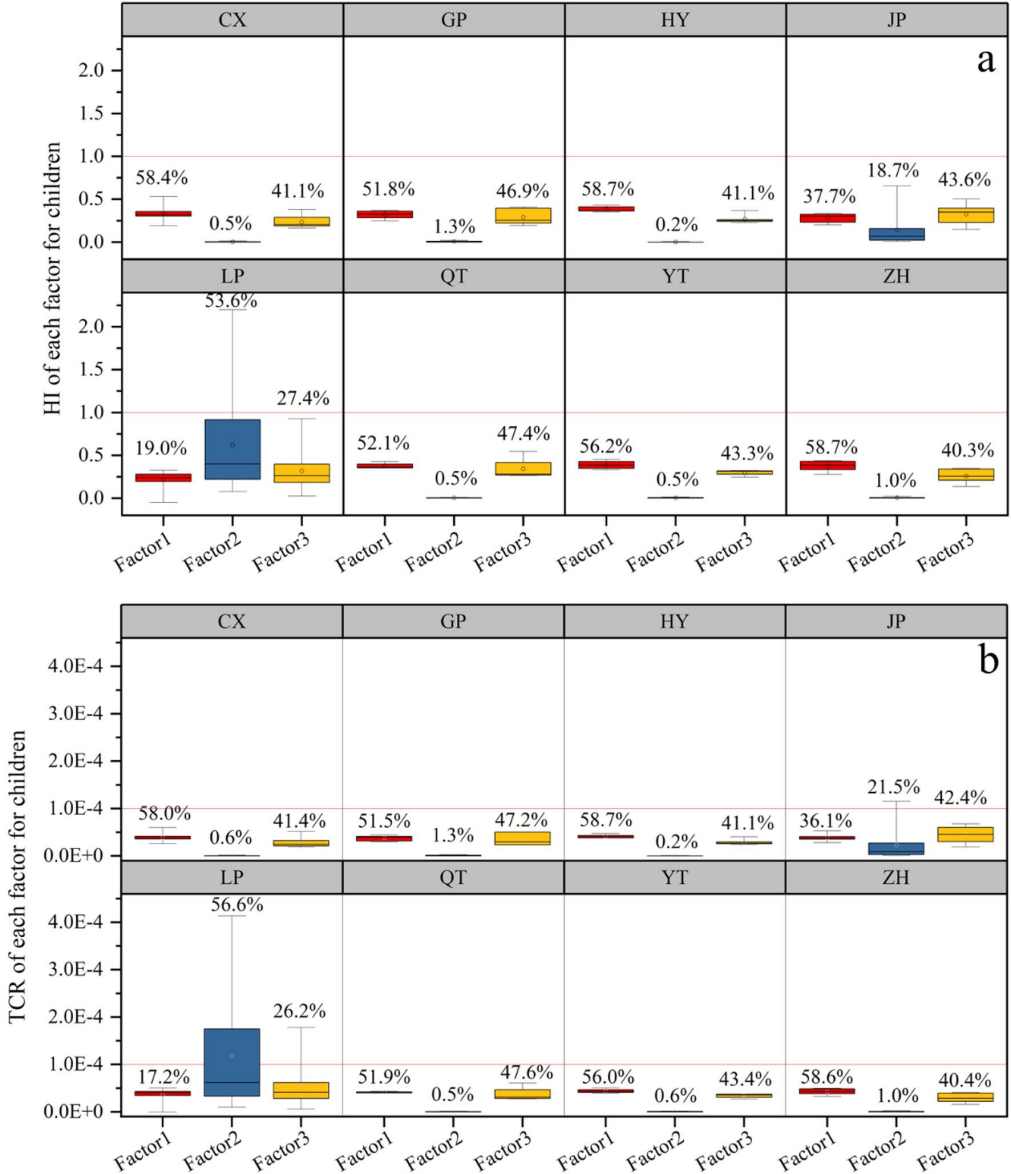
Contributions of various sources to HI and TCR for children. The red horizontal reference lines represent the corresponding threshold of HI (1) and TCR (1.0 × 10^–4^). Boxes represent the interquartile range (i.e., 25th and 75th percentiles), the hollow circles represent the average values, and the whiskers represent the 5th and 95th percentiles.

A PMF-based HR model was applied to quantitatively characterize the human health risks (non-carcinogenic risk and carcinogenic risk) of exposure to soil HMs from various sources, as shown in Fig. 6a. In the eight villages, the mean HI values of the three factors were below the threshold of 1, indicating acceptable non-carcinogenic risks of these three factors for children in these villages (except for a few samples in village LP). Besides, the three factors showed a similar order in their contribution of HMs to the non-carcinogenic risk (HI) in villages CX, GP, HY, QT, YT, and ZH, with a decreasing ranking of F2 (0.2‒1.3%) < F3 (40.1‒47.4%) < F1 (51.8‒58.7%). In addition, the contributions to the non-carcinogenic risks of the three factors in villages JP and LP were F2 (18.7%) < F1 (37.7%) < F3 (43.6%), and F1 (19.0%) < F3 (27.4%) < F2 (53.6%), respectively. There were some hotspots in village LP, where the HI for children exceeded 1. Hence, local children might suffer from the adverse non-carcinogenic risks due to HMs via F2. As Fig. 6b, the contributions of F1, F2, and F3 to TCR were similar to the HI values in the eight villages. The mean TCR values of F1, F2, and F3 in all villages (except village LP) were less than the threshold of 1.0 × 10^–4^, suggesting that the carcinogenic risks of these three factors in these villages were acceptable. Notably, the mean TCR value of F2 (1.2 × 10^–4^) in village LP surpassed the threshold, indicating that farmland HMs posed an unacceptable carcinogenic risk to children. Therefore, we should pay much more attention to F2, which represents the natural sources and originated from the high-Cd geological background of HM-rich rocks.

#### Uncertainty analysis

Undoubtedly, the successful application of the PMF model is primarily influenced by the error of the sample data, the model structure, and the parameter representation. Compared with environmental media such as the atmosphere, water, and sediment, the analysis of the sources of HMs in soils has the following three characteristics: (1) The limited migration and diffusion of HMs in soils resulted in spatial heterogeneity, which cannot strictly meet the assumption of an HM mass balance between the receptor model and source. (2) The different background contents of HMs in soils might also lead to a failure to strictly conform to the assumptions of the model. (3) The reliability of PMF mode is highly sensitive to outliers of HM dataset^3,19^. As shown in Table 1, slightly positive skewnesses of HMs concentrations were observed, indicating that outliers might exist, which might lead to uncertainty in the corresponding HM sources because the PMF model will preferentially fit the outliers to optimize the objective function Q^19^. Another uncertainty is that there is no standard rule for defining the appropriate number of factors in a PMF model^57,58^. Therefore, it remains necessary to develop the source apportionment approaches to implement risk control better.

## Conclusions

In this study, potential risks and sources of HM in farmland were investigated using a CORA/SORA approach coupling with an HDXRF spectrometer. The integrated approach provided a high-efficiency, environmentally friendly, and low-cost method for investigating pollution status and potential risk of HMs in farmland. It also clarified the HM pollutants and sources at a quantitative level. The CORA results show that Cd in farmland soils near village LP posed a “very high” potential ecological risk for the surrounding environment. The non-carcinogenic risk of HMs to children was unacceptable in villages JP and LP. The carcinogenic risk to children in village LP exceeded the threshold and had an unacceptable risk level. Among these HMs, soil Cr contributed the most the non-carcinogenic risk and carcinogenic risk to children. The SORA results indicate that F2, ascribed to the weathering of HM-rich rocks in geologically anomalous areas, should be paid much greater attention in village LP. The highest contributor to PER, HI, and TCR in village LP was F2. It is worth noting that the TCR from F2 surpassed the threshold with an unacceptable carcinogenic risk level. Consequently, HM pollutants (Cd and Cr) and sources (the geological anomaly) should be considered for risk control in this area. In general, the integrated approach combining risk assessments with the HDXRF spectrometer is effective in identifying HM pollutants and sources. It provides a valuable and excellent methodological reference for the prevention and control of HM-contaminated farmland.

## Supplementary Information

Supplementary Information.

## Data Availability

The datasets used and/or analysed during the current study available from the corresponding author on reasonable request.
