# Supplementary material for: Identification of heavy metal pollutants and their sources in farmland: an integrated approach of risk assessment and X-ray fluorescence spectrometry
Source: Sci Rep. 2022 Jul 16;12:12196. doi: 10.1038/s41598-022-16177-4 (PMC9288480; doi:10.1038/s41598-022-16177-4)
Supplement: Supplementary file 1 — Supplementary Information. [file 41598_2022_16177_MOESM1_ESM.docx]

**Supplementary information for**

Identification of heavy metal pollutants and their sources in farmlands: An integrated approach of risk assessment and X-ray fluorescence spectrometry

Xiaosong Tian ^a, ※^, Qing Xie ^b^, Min Fan ^c^, Guanqun Chai ^d^, Guanghui Li ^c^

^a^ College of Resources and Safety, Chongqing Vocational Institute of Engineering, Chongqing 402260, China

^b^ Interdisciplinary Research Center for Agriculture Green Development in Yangtze River Basin, College of Resources and Environment, Southwest University, Chongqing 400715, China

^c^ Chongqing Engineering Research Center for Soil Contamination Control and Remediation, Chongqing 400067, China

^d^ Institute of Soil and Fertilizer, Guizhou Academy of Agricultural Sciences, Guiyang 550006, China

^※^ Corresponding Authors:

Xiaosong Tian

Email: [terrytian1985@hotmail.com](mailto:terrytian1985@hotmail.com)

Address: College of Resources and Safety, Chongqing Vocational Institute of Engineering, Chongqing 402260, China.

**Main contents**: Figures, Tables and References.

**Figures**

**
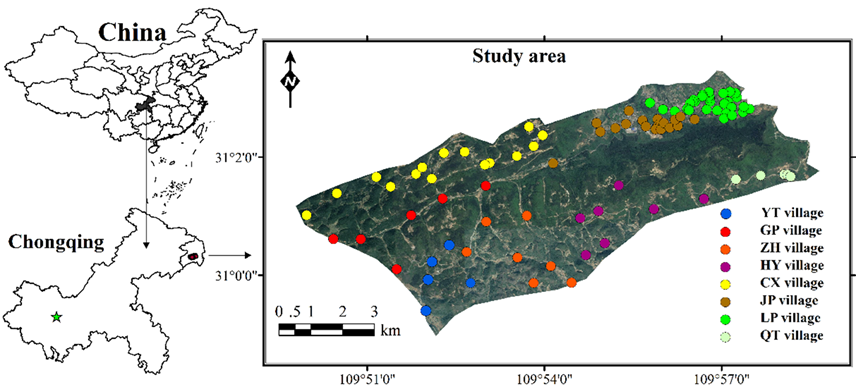
**

Figure S1 The area location and sampling distribution map. The map was created by the one of the authors (Xiaosong Tian) in ArcGIS version 10.0 (<http://www.esri.com/>).

**Tables**

Table S1 Recoveries and precisions of heavy metals in GSS4 and GSS5 via HDXRF spectrometer, and HMs detection limits（LOD）of HDXRF spectrometer ^1^

| Elements | LOD (mg/kg) | GSS4 | | GSS5 | |
| --- | --- | --- | --- | --- | --- |
|  |  | Recovery | RSD | Recovery | RSD |
| Cr | 16 | 108.37% | 4.13% | 94.82% | 4.61% |
| Ni | 4 | 108.87% | 3.23% | 91.16% | 3.48% |
| Cu | 0.8 | 108.60% | 3.63% | 107.34% | 4.98% |
| Zn | 1.6 | 97.36% | 3.16% | 104.27% | 3.42% |
| As | 0.8 | 97.73% | 2.67% | 103.91% | 1.49% |
| Cd | 0.09 | 95.45% | 7.65% | 105.88% | 6.77% |
| Pb | 0.8 | 92.48% | 0.92% | 100.34% | 1.31% |

LOD represents HMs detection Limit of the instrument; RSD represents the relative standard deviation.

Table S2 Classification criterion of the potential ecological risk index

| $\text{E}_{\text{r}}^{\text{i}}$ | The potential ecological risk for single heavy metal | PER | The potential ecological risk for all heavy metals |
| --- | --- | --- | --- |
| $\text{E}_{\text{r}}^{\text{i}}$ < 40 | None | *PER* < 150 | None |
| 40 ≤ $\text{E}_{\text{r}}^{\text{i}}$ < 80 | General | 150 ≤*PER* < 300 | General |
| 80 ≤ $\text{E}_{\text{r}}^{\text{i}}$ < 160 | Moderate | 300 ≤*PER* < 600 | Moderate |
| 160 ≤ $\text{E}_{\text{r}}^{\text{i}}$ < 320 | High | 600≤ *PER<* 1200 | High |
| $\text{E}_{\text{r}}^{\text{i}}$ ≥ 320 | Very high | *PER*＞1200 | Very high |

Table S3 Parameters for exposure dose assessment for children ^2,3^.

| Parameter | Description | Unit | Value |
| --- | --- | --- | --- |
| IR_ing_ | Daily ingestion rate of soil | mg/day | 200 |
| IR_inh_ | Daily inhalation rate of soil | m3 /day | 7.6 |
| SA | Skin area available for soil contact | cm^2^ | 2800 |
| AF | Soil-to-skin adherence factor | kg/( cm^2^ ×day) | 1E-06 |
| ABS | Absorption factor for As | unitless | 0.03 |
| ABS | Absorption factor for others | unitless | 0.001 |
| PEF | Particle emission factor | m^3^ /kg | 1.36E+09 |
| EF | Exposure frequency | day /year | 350 |
| ED | Exposure duration | year | 6 |
| BW | Body weight | kg | 29 |
| AT | Average time for non-carcinogens | day | 2190 |
| AT | Average time for carcinogens | day | 25550 |

Table S4 Reference doses (mg/(kg·day)) and slope factors (per mg/(kg·day)) for seven heavy metals ^2,3^.

| Parameter | Cd | Cr | As | Pb | Cu | Zn | Ni |
| --- | --- | --- | --- | --- | --- | --- | --- |
| RfD for ingestion | 1.00E-03 | 3.00E-03 | 3.00E-04 | 3.50E-03 | 4.00E-02 | 3.00E-01 | 2.00E-02 |
| RfD for dermal contact | 1.00E-05 | 6.00E-05 | 1.23E-04 | 5.25E-04 | 1.20E-02 | 6.00E-02 | 5.40E-03 |
| RfD for inhalation | 1.00E-05 | 2.86E-05 |  |  |  |  | 9.00E-05 |
| SF for ingestion | 6.10E+00 | 5.00E-01 | 1.50E+00 | 8.50E-03 |  |  | 1.70E+00 |
| SF for dermal contact |  |  | 3.66E+00 |  |  |  |  |
| SF for inhalation | 6.30E+00 | 4.20E+01 | 1.50E+01 |  |  |  | 8.40E-01 |

Table S5 Risk screening value (RSV) recommended by the *National Environmental Quality Standards of China* (GB15618-2018)*.*

| Pollutants | Land use types | Risk screening value (RSV) | | | |
| --- | --- | --- | --- | --- | --- |
|  |  | pH≤5.5 | 5.5＜pH≤6.5 | 6.5＜pH≤7.5 | pH＞7.5 |
| Cd | Others land-use type | 0.3 | 0.3 | 0.3 | 0.6 |
| As | Others land-use type | 40 | 40 | 30 | 25 |
| Pb | Others land-use type | 70 | 90 | 120 | 170 |
| Cr | Others land-use type | 150 | 150 | 200 | 250 |
| Cu | Others land-use type | 50 | 50 | 100 | 100 |
| Ni | —— | 60 | 70 | 100 | 190 |
| Zn | —— | 200 | 200 | 250 | 300 |

Table S6 Summary of PMF and error estimation diagnostics for the base run of 2, 3, and 4 factors.

| Diagnostic | 2 factors | 3 factors | 4 factors |
| --- | --- | --- | --- |
| *Q*_expected_ | 436 | 339 | 242 |
| *Q*_ture_ | 32824 | 14403 | 6133 |
| *Q*_robust_ | 7446 | 4105 | 2394 |
| *Q*_robust_/*Q*_expected_ | 17 | 12 | 10 |
| DISP % dQ (%) | <0.1% | <0.1% | <0.1% |
| DISP swaps | 0 | 0 | 0 |

Table S7 The fitted results of the heavy metals concentrations by PMF.

| Species | Category | S/N | Intercept | Slope | SE | R^2^ | KS Test | |
| --- | --- | --- | --- | --- | --- | --- | --- | --- |
|  |  |  |  |  |  |  | Stat | P-Value |
| Cr | Strong | 8.80 | 14.65 | 0.88 | 78.60 | 0.90 | 0.28 | 0.00 |
| Cu | Strong | 9.67 | 10.76 | 0.77 | 11.05 | 0.72 | 0.22 | 0.00 |
| As | Strong | 9.78 | 8.96 | 0.47 | 1.68 | 0.54 | 0.12 | 0.06 |
| Cd | Strong | 8.15 | 0.12 | 0.97 | 1.00 | 0.98 | 0.35 | 0.00 |
| Pb | Strong | 9.95 | -2.29 | 1.07 | 3.26 | 0.51 | 0.16 | 0.00 |
| Ni | Strong | 9.66 | 3.38 | 0.95 | 9.67 | 0.96 | 0.15 | 0.01 |
| Zn | Strong | 9.86 | 19.64 | 0.81 | 35.23 | 0.86 | 0.17 | 0.00 |

**References**

1 Tian, X. *et al.* Risk Identification of Heavy Metals in Agricultural Soils from a Typically High Cd Geological Background Area in Upper Reaches of the Yangtze River. *Bulletin of environmental contamination and toxicology*, doi:10.1007/s00128-021-03417-y (2022).

2 Wu, J., Li, J., Teng, Y. G., Chen, H. Y. & Wang, Y. Y. A partition computing-based positive matrix factorization (PC-PMF) approach for the source apportionment of agricultural soil heavy metal contents and associated health risks. *Journal of hazardous materials* **388**, 121766-121766, doi:10.1016/j.jhazmat.2019.121766 (2020).

3 Yang, S. *et al.* An integrated analysis on source-exposure risk of heavy metals in agricultural soils near intense electronic waste recycling activities. *Environment International* **133**, 105239, doi:10.1016/j.envint.2019.105239 (2019).
